# Heterogeneous virulence of pandemic 2009 influenza H1N1 virus in mice

**DOI:** 10.1186/1743-422X-9-104

**Published:** 2012-06-06

**Authors:** Amber Farooqui, Alberto J Leon, Yanchang Lei, Pusheng Wang, Jianyun Huang, Raquel Tenorio, Wei Dong, Salvatore Rubino, Jie Lin, Guishuang Li, Zhen Zhao, David J Kelvin

**Affiliations:** 1 Division of Immunology, International Institute of Infection and Immunity, Shantou University Medical College, 22 Xinling Road, Shantou, Guangdong, 515041, China; 2 Department of Biomedical Sciences, University of Sassari, Sassari, Italy; 3 Division of Experimental Therapeutics, Toronto General Research Institute, University Health Network, 101 College Street, Toronto, ON, M5G 1 L7, Canada; 4 Division of Viral Hepatitis and Liver Failure, Infectious Disease Hospital, Nanchang University, Nanchang 9th Hospital, 167 Hongdu Middle Road, Nanchang, Jiangxi, 330002, China; 5 Center for Disease Control and Prevention of Shantou, 58 Shanfen Road, Shantou, Guangdong, 515041, China; 6 Center for Biotechnology Development and Biodiversity Research, University of Sassari, Sassari, Italy

**Keywords:** Pandemic H1N1 influenza, Viral heterogeneity, Clinical presentation, Host adaptation, Viral polymerase, Virulence, Pathogenesis

## Abstract

**Background:**

Understanding the pathogenesis of influenza infection is a key factor leading to the prevention and control of future outbreaks. Pandemic 2009 Influenza H1N1 infection, although frequently mild, led to a severe and fatal form of disease in certain cases that make its virulence nature debatable. Much effort has been made toward explaining the determinants of disease severity; however, no absolute reason has been established.

**Results:**

This study presents the heterogeneous virulence of clinically similar strains of pandemic 2009 influenza virus in human alveolar adenocarcinoma cells and mice. The viruses were obtained from patients who were admitted in a local hospital in China with a similar course of infection and recovered. The A/Nanchang/8002/2009 and A/Nanchang/8011/2009 viruses showed efficient replication and high lethality in mice while infection with A/Nanchang/8008/2009 was not lethal with impaired viral replication, minimal pathology and modest proinflammatory activity in lungs. Sequence analysis displayed prominent differences between polymerase subunits (PB2 and PA) of viral genomes that might correlate with their different phenotypic behavior.

**Conclusions:**

The study confirms that biological heterogeneity, linked with the extent of viral replication, exists among pandemic H1N1 strains that may serve as a benchmark for future investigations on influenza pathogenesis.

## Background

Following the emergence of the initial few cases in Mexico and California in 2009, the world faced another episode of pandemic caused by the novel influenza A H1N1 virus (pdm H1N1 hereafter) that carried a unique combination of gene segments from four different lineages [1]. The virus spread so rapidly that within two months of the first confirmed report, the World Health Organization (WHO) declared a level VI global emergency alert. Epidemiologic observations affirm the presence of seasonal flu imprints in pandemic H1N1 strains such as high attack rate with mild presentation and self-limiting infection in the majority of human cases [2]; however, some of them led to severe respiratory illness and eventually death [3,4]. Absence of known virulence markers such as lysine (K) residue at 627 in PB2 and the multi-basic cleavage site in hemagglutinin (HA), as well as truncated PB1-F2 and NS1 proteins [1], support the modest morbidity profile of pandemic H1N1 viruses. In addition, several in vivo studies conducted in ferrets and mice confirm the subtle disease profile due to pdm H1N1 despite its efficient replication in the lower respiratory tract of the host coupled with increased levels of innate and adaptive immune mediators [5-7]. However, severe and fatal human cases are reasonably explained by the presence of underlying host illness and bacterial co-infections that dysregulate host immune functions and consequently weaken host’s ability to control viral replication [8-10]. One can consider the important role of host-associated factors in disease outcome, the reason pdm H1N1 behaved differently in humans remains elusive. Recently, Safronatz *et al.* reported the diversified behavior of pdm H1N1 strains of Mexican origin in cynomolgus macaques that indicates the possible link between viral heterogeneity and degree of disease severity[11].

**Table 1:** Genetic, demographic and Clinical background of 2009 pdm H1N1 influenza strains

		**A/Nanchang/8002/2009** **(NC2)**	**A/Nanchang/8008/2009** **(NC8)**	**A/Nanchang/8011/2009** **(NC11)**
Day of sample collection		9 Dec 2009	19 Dec 2009	22 Dec 2009
Age (yrs)		27	15	33
Gender		M	F	F
Temperature (^o^C)		39.4	38.5	NA
Hospitalization		✓	✓	✓
Complications				NA
Antiviral Treatment		✓		
Lethality in mice		Severe	Mild	Severe
Viral loads in mice lungs		High	Low	High
Replication in A549 cells		High	Moderate	High
^£^TCID_50_/ml		1 x 10^4.83^	1 x 10^4.33^	ND
EID_50_/ml		1 x 10^6.4^	1 x 10^7.75^	1 x 10^6.4^
^€^PB2	Genbank ID	JF800142	CY089613	CY089621
	V227I		✓	
	V295L			✓
	R299K		✓	
	I310R			✓
	K353R			✓
	N556K			✓
	T588I			✓
	K660R	✓		
^€^PB1	Genbank ID	JF800143	CY089614	CY089622
	T257A	✓		
^€^PA	Genbank ID	CY089607	CY089615	CY089623
	A70V		✓	✓
	P224S	✓	✓	✓
	E243G		✓	
	E319V	✓		
	D547E			✓

^£^Observed in MDCK cells, NA: not available, ND: not detected, ^€^Mutations are compared with prototypic strain: A/California/07/2009 H1N1.

Several laboratory animals including mice, ferrets, cotton rats and nonhuman primates have been successfully used as suitable models of influenza infection [12]. Among them ferrets are considered the best because of their natural susceptibility to the virus and its similar pathogenesis to that of humans [13]; however, their use in large-scale screening is not feasible. Small laboratory animals, particularly mice, have shown promising potential for virological studies. We have previously described the infection of prototypic pdm H1N1 strain, A/ Mexico/4108/2009 in mice with significant viral replication and marked lung pathology [14].

The high magnitude of the 2009 pandemic and potential risk of future outbreaks necessitate the evaluation of newer viral strains to resolve ambiguities about the severity of infection. In this study, we evaluated three different H1N1 influenza viruses that were isolated from adult patients admitted in a local hospital in the southern part of China during the second pandemic wave. Interestingly, these strains exhibited mild to severe pathogenic potential in terms of viral replication, disease progression, and induction of proinflammatory response *in vitro* and *in vivo.* Sequence analysis reveals that the mutations in polymerase subunits (PB2 and PA) might correlate with the phenotypic trait of the viruses. This study presents the co-circulation of heterogeneous pdm H1N1 during this period that cannot be a neglected factor in evaluating the pathogenesis of 2009 pandemic influenza infection.

## Results

### Differential response of pdm H1N1 strains in A549 cells

**Figure 1:**
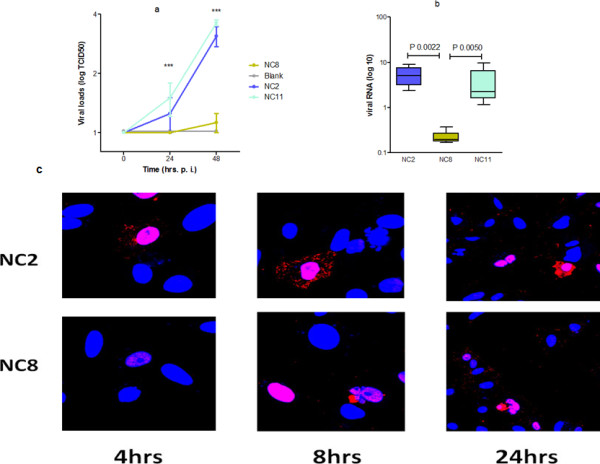
**Comparison of viral replication kinetics in A549 cells.** A549 cells were infected with viral strains; NC2, NC8 and NC11 at MOI 2. **(a**) shows the viral titers in cell supernatants 24 and 48 h post infection (*P* < 0.0001). (**b**) shows viral mRNA levels in infected cells and (**c**) Nuclear localization of viral NP protein was noticeably slow in cases of NC8 infection at 4 h that led to no or negligible yield of viral progeny in cell cytoplasm at 8 h post infection. Red shows staining of viral NP protein and blue shows nucleus specific DAPI stain. Micrographs are taken at the magnification of 40 (for 4 h and 8 h) and at 20 for 24 h.

To better understand the pathogenesis profile of local isolates, we randomly selected three strains of pdm H1N1, namely A/Nanchang/8002/2009 (NC2), A/Nanchang/8008/2009 (NC8), and A/Nanchang/8011/2009 (NC11). All were isolated from adult patients with severe clinical profiles and without underlying illnesses (Table 1). First we evaluated the replication and inflammatory response of these strains in human adenocarcinoma alveolar epithelial (A549) cells that were inoculated with each viral strain at multiplicity of infection (MOI) 2. Out of three, two pdm H1N1 strains (NC2 and NC11) exhibited severe cytopathic effects while, unexpectedly, NC8 caused only mild infection in A549 cells. Viral titration of culture supernatants was performed in MDCK cells at different time points. Kinetics studies showed that NC2 and NC11 grew in high titers in 24 h which was further increased in 48 h while NC8 showed poor replication capacity throughout the study period (*P <* 0.0001) Impaired replication of NC8 was also verified by significantly lower viral mRNA and protein levels over the period of 24 h post infection. Confocal laser fluorescent microscopy showed that NC8 was capable of infecting cells but replication was delayed compared to that of NC2 infection (Figure 1)

**Figure 2:**
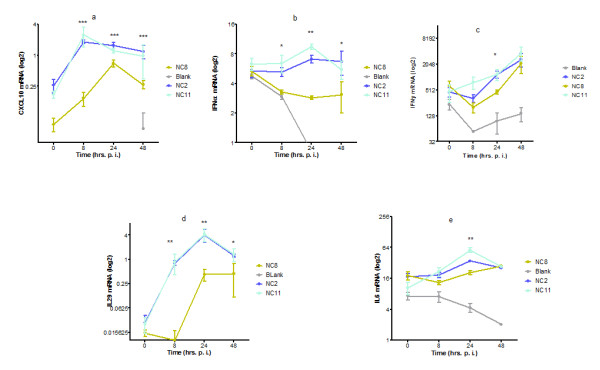
**Comparison of infection with different strains of pdmH1N1 virus in A549 cells.** The figure shows the comparative analysis of cellular immune mediators after NC2, NC8 and NC11 infection. Cells were infected with respective viral strains while mock infection with HBSS was given to blank. At different time points, mRNA expression of different cytokine/chemokine genes was measured by real time RT-PCR. Results are presented as log 2 of mean arbitrary units ± SEM. Data are compared by Mann Whitney U test. *** _*P* < 0.0001, **_ *P* <0.001, * _ *P* <0.01.

Transcriptional analysis of major inflammatory mediators was performed by real time PCR at four different time points over the period of 48 h post infection. We observed that NC8 was unable to mount efficient inflammatory response as a consequence of its poor replication. As shown in Figure 2, CXCL10 expression was mute in cases of NC8 infection compared with NC2 and NC11 throughout the study period (*P* < 0.0001). In addition, cellular interferon responses including interferon (IFN) γ, IFN αA2 (*P* < 0.0001), and IL29 (*P* <0.0068) were also weak after NC8 infection compared with NC2 and NC11; however, in the case of IFN- γ, significant difference was observed only in 24 h (*P* < 0.05). A similar difference was observed in the case of IL6 at 24 h post infection (*P* 0.0176). Conclusively, immune mediators peaked between 8 to 24 h after NC2 and NC11 infection, while minimal variations in the gene expression were observed in NC8-infected cells.

The above-mentioned results indicated relatively poor replication ability of the NC8 strain in mammalian cells coupled with weak inflammatory response that prompted us to scrutinize how NC8 behaves in an avian environment. In contrast with A549 cells, a different situation was observed in chicken embryo nated eggs in which the virus titers of NC8 were higher than those from NC2 and NC11 (Table 1).

### Differential pathogenesis of pdmH1N1 strains in mice

**Figure 3:**
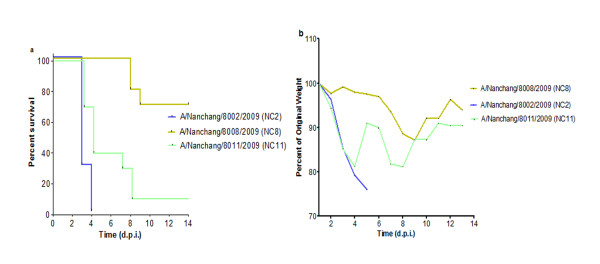
**Comparison of the virulence of three pandemic H1N1 strains in C57/BL6 mice.** Viral strains NC2, NC8 and NC11 were analyzed in C57/BL6 mice (n = 10/group). Viral infection was established with 10^5^ EID_50_ of NC2, NC11 and 10^6^ EID_50_ of NC8 and animals were observed up to 14 days. (**a**) Kaplan Meier’s analysis shows overall difference in survival curves (*P* 0.0007). Log-rank sum test shows differences between mortality curves: NC2 vs NC8 (*P* < 0.0001), NC2 vs NC11 (*P* 0.0195) and NC8 vs NC11 (*P* 0.0007). (**b**) Weight loss curve for the group of animals infected with NC2, NC8 and NC11. Significant differences were observed at 3 d.p.i., NC8 vs NC2 (*P* < 0.0001), NC8 vs NC11 (0.0007).

We next evaluated the pathogenesis of NC2, NC8 and NC11 viruses in C57/BL6 mice (Figure 3). Each group of animals was infected with the same dose of influenza virus intranasally and observed for weight loss and lethality up to 14 days. Most strikingly, these viral strains, with apparently the same clinical profile in humans, behaved differently in C57/BL6 mice in lethality (*P* 0.0007) and weight loss (*P* 0.0007). When using 20% weight loss as the humane point, NC2 was 100% lethal within four d.p. i. at 10^5^ EID_50_. At the same viral dose, NC11 exerted 90% lethal response within eight d.p.i., although no significant difference was noted in median death day (MDD), number of survivors and weight loss kinetics between the two viral strains. On the other hand, the same infection dose of NC8 did not cause death in animals while the virus at 10-fold concentration (10^6^ EID_50_), resulted only in 30% lethality (*P* < 0.001) coupled with a significant dichotomy in clinical course of infection within the NC8 infected group. Weight losses were milder and delayed as compared with those of the other groups (*P* < 0.001). The weight loss kinetics of individual animals infected by each virus is given in additional file 1. Taken together, these wild type pdm H1N1 strains showed heterogeneous attitude in C57/BL6 mice.

**Figure 4:**
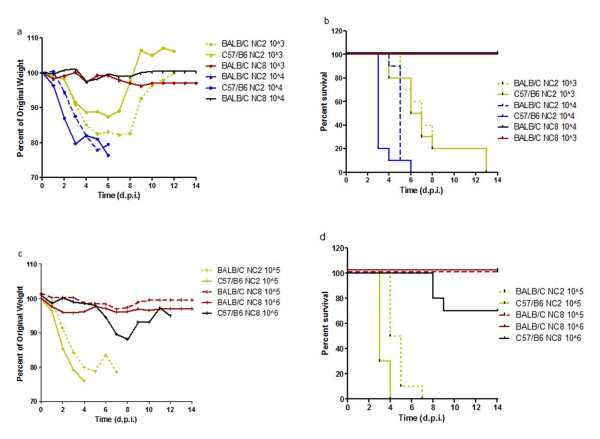
**Analysis of biological heterogeneity in different mice strains in dose dependent manner.** Pandemic H1N1 viruses (NC2 and NC8) were compared in C57/BL6 and BALB/C mice in dose dependent manner. Weight losses (**a, c**) and lethality (**b, d**) are shown for animals (n = 10/group) infected with varying concentrations of viral strains (10^3^, 10^4^, 10^5^ and 10^6^). Log rank sum test shows significant differences in mortality curves between NC2 and NC8 irrespective of inocula and mice strain (P < 0.0001). ). (**a, c**): Inter-group comparisons in weight loss shows significant difference NC2 vs NC8 groups (P < 0.0001), C57/BL6 vs BALB/C; NC2_ 10^4^ (*P* < 0.0001), NC2_10^5^ (*P* < 0.0001). (**b, d**): Survival differences are also significant in C57/BL6 vs BALB/C after NC2 infection: 10^4^ (*P* < 0.0028), 10^5^ (*P* 0.0005), 10^6^ (*P* 0.0669).

To confirm the heterogeneous nature of NC2 and NC8, viral infection was further established in C57/BL6 and BALB/C mice in a dose dependent manner. In addition, comparisons between the survival curves of C57/BL6 and BALB/C were analyzed since different mouse strains might differ in susceptibility of wild type strains. C57/BL6 mice appeared to be more susceptible to viral infection than BALB/C; however, the trend was clearer at low viral doses. In general, NC2 infected mice faced 100% lethality irrespective of viral dose; however, they exhibited dose dependent kinetics in weight loss and survival curve with an extension of MDD from days 3 to 7 at high (10^5^ EID) and low viral inocula (10^3^ EID) respectively. In contrast, NC8 was unable to mount lethal infection irrespective of viral inocula and animal strains except 30% lethality with modest weight loss in C57/BL6 mice infected with 10^6^ EID_50_ (Figure 4). These observations clearly demonstrate the presence of two different virulence phenotypes of pdm H1N1 strains, of which one seems to be better adapted to mammalian hosts than the other.

### Altered replication of viral strains in mice

**Figure 5:**
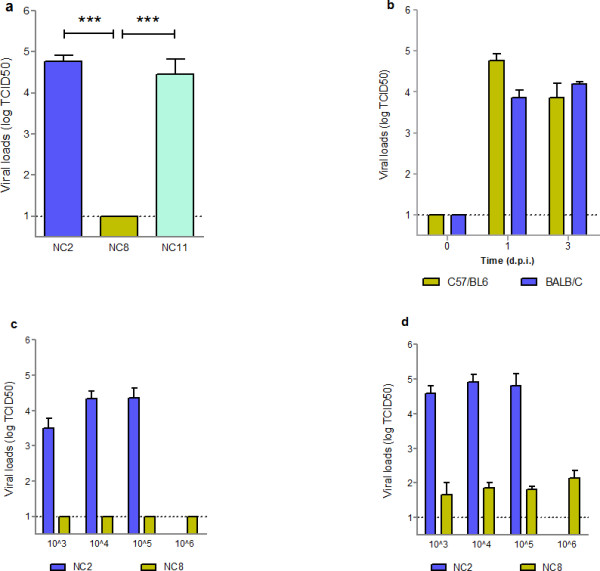
**Heterogonous viral replication of pandemic influenza H1N1 strains in mice lungs.** (**a**) Viral loads in the lungs of NC2, NC8 and NC11 infected C57/BL6 mice were determined at 1 d.p.i.. (**b**) Viral loads NC2 were also examined in C57/BL6 and BALB/C mice (n =3). Replication kinetics of NC2 and NC8 were compared in BALB/C mice infected with dose series of viruses at (**c**) 1 d.p.i. and (**d**) 3 d.p.i.. MDCK cells were used to determine viral loads in serially diluted lung homogenates collected at different time intervals. Results are presented as mean ± SEM of TCID_50_. Statistical differences were calculated by Mann Whitney U test. *** - *P* < 0.0001.

We also determined the replication and organ distribution (viral loads) of pdm H1N1 strains in different animal body tissues by cell culture method. Lung homogenates of NC2 and NC8 infected (10^5^ EID_50_) C57/BL6 mice mimic the viral replication profile of A549 cells at 1 d.p.i. with slightly enhanced viral titers of NC11 (Figure 5a); however, all three viral strains grew at the same rate at 3 d.p.i. (data not shown). The results indicate delayed replication of NC8 that might provide a chance for the host immune system to overcome the infection, which eventually results in a non-lethal infection. Comparison of viral replication of NC2 in lungs of BALB/C and C57/BL6 mice showed that NC2 at 10^5^ EID_50_ replicated well in both mouse strains. To further understand the growth pattern of NC2 and NC8 strains, we titrated lung homogenates of BALB/C mice infected with dose series of the viruses. We found that NC8 was not able to replicate at day 1. Although day 3 showed signs of NC8 replication in animal lungs, the viral titers were still significantly lower than those of NC2 (*P* < 0.002) (Figure 5). No extrapulmonary viral spread was evidenced. The results endorsed the above observations and confirmed that early viral replication contributes to the pathogenesis of pandemic H1N1 infection.

### Lung pathology

**Figure 6:**
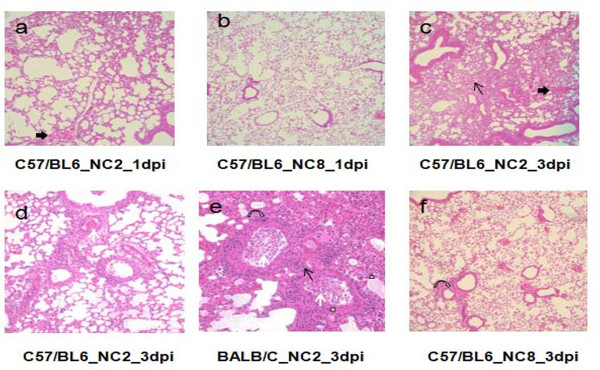
**Lung pathology of mice infected with different viral strains.** The figure shows lung pathology of C57/BL6 mice at 1 d.p.i. (**a**) and 3d.p.i. (**c and d**) and BALB/C at 3 d.p.i. (**e**) after NC2 infection. In contrast NC8 infected C57/BL6 mice show no (**b**) and very mild (**f**) pathological signs at 1 and 3 d.p.i. respectively. Thin arrows – inflammation in interstitial space; thick arrows – hemorrhage in respiratory bronchioles; white arrows – infiltrating cells in lumen of terminal bronchiole; curved arrows – inflammation in peribronchial area; white triangle – pulmonary edema; white cross – damaged epithelium.

The extent of alveolar damage caused by NC2 and NC8 was assessed by histology over time. Figure 6 presents the comparison of haematoxylin and eosin (H&E) stained infected lung tissues. In C57/BL6 mice, NC2 caused mild to moderate cellularity in interstitial space at 1 d.p.i. compared with NC8, which did not show any sign of inflammation (Figure 6a and b). Severe interstitial inflammation with damaged alveolar structure, moderate cellular exudates, and hemorrhage in the lumen and peribronchial spaces were noticed after three days of NC2 infection (Figure 6c and d). Alveolar edema and distortion of respiratory epithelium was also observed in NC2 infected BALB/C mice (Figure 6e). In contrast, inflammatory scores were much lower after three days of NC8 infection as shown in Figure 6f. Histological observations support the heterogeneous nature of NC2 and NC8 infection as observed by survival and viral replication experiments.

### Evaluation of host immune responses

**Figure 7:**
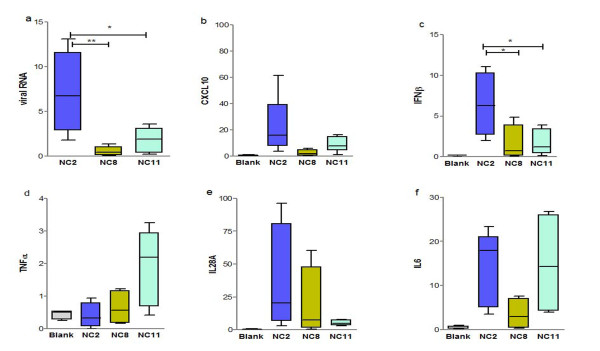
**Evaluation of host immune mediators.** Transcriptional data for major host immune response genes (**b-f**) and viral mRNA levels (**a**) are shown at 1d.p.i. in C57/BL6 mice infected with 10^5^ EID_50_ of various strains of pandemic H1N1 influenza virus (NC2, NC8 and NC11) and mock infection with HBSS was given to blank animals. Results are presented as mean ± SEM of mRNA levels normalized with mouse β-actin gene. Statistical differences were calculated by Mann Whitney U test. ** - *P* < 0.001, * - *P* < 0.01.

First we compared the ability of NC2, NC11 and NC8 viruses to induce host inflammatory immune response in animals. C57/BL6 mice were infected with equal amount of viruses while mock infection with HBSS was administered to the blank group. We also noted whether weight loss and viral replication patterns were similar to those in the above-mentioned experiments. As expected, the gene expression of major immune mediators including CXCL10, IFNβ, TNFα, IL29 and IL6 of NC2 and NC11 infected mice was more pronounced than in those who were infected with NC8. At 1 d.p.i., the expression of major inflammatory mediators CXCL10 and IFN β was upregulated in NC2 and NC11 infected groups, while the highest increase in the expression of TNFα and IL28A was observed in NC11 and NC2 respectively (Figure 7). On the other hand, transcriptional analysis of NC8 infected C57/BL6 mice showed attenuated response compared with NC2 and NC11 (*P* < 0.0001), mimicking the data obtained from A549 cells probably due to inefficient viral replication.

On the basis of the above-mentioned data, we chose the NC2 infection model to compare the kinetic response in BALB/C and C57/BL6 mice by real time RT-PCR analysis. More robust gene expression was observed in C57/BL6 mice compared with BALB/C, who showed comparatively attenuated responses throughout the experiment. In C57/BL6 mice, proinflammatory response was marked with significant induction of early immune mediators such as CXCL10, IFN β and TNFα. The trend was similar for IL6, indicating the classical switching of innate and adaptive arms. The highest level of expression was achieved at 1 d.p.i. in each case with the exception of IL28A, which was progressively increased over time (additional file 2).

### Genetic characterization

Whole viral genomes of these strains were further sequenced to evaluate genetic mutations that might explain their biological behavior in cells and mice. Sequences were deposited to Genbank [Genbank: JF800142, JF800143, CY089607, CY089608, CY089609, CY089610, CY089611, CY089612] for A/Nanchang/8002/2009 (NC2), [Genbank: CY089613, CY089614, CY089615, CY089616, CY089617, CY089618, CY089619, CY089620] for A/Nanchang/8008/2009 (NC8) and [Genbank: CY089621, CY089622, CY089623, CY089624, CY089625, CY089626, CY089627, CY089628] for A/ Nanchang/8011/2009 (NC11) strain.

Several mutations were found in each gene segment with respect to prototypic pdm H1N1 strains, A/California/07/2009 and A/California/04/2009. Comparison between NC2, NC8 and NC11 genomes revealed that NC8 differed from NC2 and NC11 at three different positions in polymerase subunits PB2-V227I, R299K and PA-E243G. HA analysis also showed the substitution of alanine at position 409 in NC2 and NC11 which was not present in NC8 (Table 1). Experimental data have already shown that NC2 and NC11 are more virulent than NC8 due to efficient viral replication; possibly these amino acid residues in PB2, PA and HA gene have an important role in host adaptation and the virulence of pdm H1N1 influenza virus. However, additional studies are required to probe the biological relevance of these amino acid changes.

Genetic characterization of NA, PB1, NP, NS1 and M2 also showed various mutations in these segments; however, none of them clearly defined the different pathogeneses of these pdm H1N1 strains (additional file 3).

## Discussion

Here we present the heterogeneous virulence of three different strains of influenza H1N1 in human adenocarcinoma cells (A549) and mice that were isolated from clinically similar human cases from South China in December 2009. Two different patterns of biological heterogeneity were observed: first, two strains (NC2 and NC11) showed efficient viral replication and subsequent effects on tissue histology, induction of proinflammatory response and causing lethality in mice, although their behavior was not totally identical and some minor differences in the kinetics of the disease in mice were observed. Secondly, NC8 showed delayed replication that eventually led to non-lethal infection and muted inflammatory response in mice. These results have a relevance to the previously published epidemiological reports that associate effective viral replication and delayed clearance with disease severity in humans [9,15,16]. Most of the previous studies agree that pdm H1N1 exert homogeneous and modest infection but with efficient pulmonary viral replication in mice [17]; however, its pathogenesis is more than that of seasonal strains [5,18] and subdued compared with 1918 pandemic and other swine origin influenza viruses [17,19]. Nonetheless, the virus has been shown to increase virulence upon expression of truncated viral proteins by reverse genetic tools and after mice adaptation [20]. In addition to the heterogeneous nature of these strains, we also demonstrate that C57/BL6 mice are more susceptible for pdm H1N1 infection than BALB/C strains; however, variation in disease kinetics did not change the infectivity ratios as observed previously [14].

In this study, NC2 and NC11 viruses were able to induce of proinflammatory cytokines effectively whereas immune responses were mute in the case of NC8 infection. It is interesting to note that pdm H1N1 strains also display a differential cytokine response which may or may not be linked with viral growth. Previous studies have shown a robust gene expression of innate immune response genes with delayed switch to adaptive immunity after pdm H1N1 infection; however, overall responses are considered to be higher compared with those of contemporary seasonal strains [7,17].

Clinically, 2009 influenza pandemic caused self recovering mild disease in the vast majority of patients while only a small group of patients developed serious respiratory complications [21,22]. No explanation has yet been offered for why the clinical profile varies from one patient to another. The published studies interrogating host markers and viral pathogenesis *in vitro* and *in vivo* are mostly limited to the characterization of a narrow range of prototypic pdm H1N1 strains [5,17-19]. Consequently, important aspects of the disease may remain unexplored. Pandemics provide a greater chance for influenza viruses to mutate; however, unveiling their impact on viral pathogenecity is an enduring goal that can be achieved by continuous surveillance. Lab investigation of newer strains might provide valuable information about the pathogenesis that could be missing in initial studies.

In the present study, although all these viral strains were isolated from patients who finally recovered, the viruses were able to produce biological heterogeneity in mice that refute the common paradigm of the evaluation of influenza pathogenesis which is at present based on the clinical profile and disease outcome of patients. Such attributes have previously been observed in clinically relevant influenza H5N1 strains [23,24]. In humans, viral heterogeneity may have specific effects on individuals with different genetic background and demography; therefore, infection with such viruses might result in a variable clinical course of infection. However, it is also important to remember that treatment strategies, immunocompetance, and clinical management influence the disease severity and outcome and consequently mask the true picture of viral pathogenesis.

Virulence and interspecies transmission of influenza virus is often considered a polygenic phenomena [25-28]. The triple reassortant pandemic 2009 influenza virus stands out from ancestral pandemic and reassorted strains because it rapidly transmits to humans despite the absence of any traditional virulence markers; for example, the C-terminal PDZ ligand domain of NS1 [26], functional PB1-F2 protein, and PB2- K627 [1]. Therefore, efforts have been made to determine other possible virulence determinant(s). Recent laboratory investigations conducted with mouse adapted pdm H1N1 strains speculate the role of HA (D131E and S186P) [29] and PB2 genes, such as glutamate-to-glycine substitution at 158 [30], aspartate to asparagines at 701 [31], Threonine-to-alanine at 271 [32] and second site suppressor mutation [33] in viral replication and mouse adaptation, although none of them was demonstrated in wild type strains. In this study, the genetic characterization showed that the non-lethal NC8 strain contained three mutations (PB2-V227I, R299K and PA-E243G) in polymerase subunits compared with virulent strains. Previous studies have reported that both PB2 and PA genes are genetically linked with each other [34]; furthermore, N-terminal mutations in these genes might lead to intermediate or complete loss of viral RNA transcription [35]. Therefore, we might speculate that these mutations are interlinked and collectively responsible for altered replication of the NC8 strain. On the other hand, this virus (NC8) strikingly replicated more than other viruses with > log10 ratio in embryonated chicken eggs, indicating the ease of growth in an avian environment. Here it is important to consider that the 2009 pandemic virus contains polymerase subunits PB2 and PA of North American avian lineage. We do not know whether these substitutions in NC8 are the remains of ancestral avian strains or not, but upon sequence analysis of global pdm H1N1 isolates, we found that these amino acid residues (PB2-I227, K299, PA-G243) are conserved in pdm H1N1 strains, thus raising the possibility that collectively they have some role in the adaptation to the mammalian host and they might link to the heterogeneity of pdm H1N1. However, *in vivo* studies with mutant strains are required to prove the hypothesis. In the case of HA gene, NC2 and NC11 contained the A409V mutation compared to NC8 and prototype California strains. It is worthwhile to indicate that NC2 also exhibited HA-E391K, which has recently been identified as a fast-growing mutation with the ability to destabilize the HA oligomerization process, thus modifying the membrane fusion properties of the pandemic influenza virus [36,37]. However, no association with virulence and progression of disease has been established yet. Taken together, we hypothesize that these mutations in PB2, PA and HA genes might have no relevance with human disease but in the case of zoonotic transmission of influenza viruses to human, it may yield more pathogenic viruses.

## Conclusions

In conclusion, the study provides evidence about the heterogeneous replication and virulence of clinically relevant pandemic influenza H1N1 viruses in mice and human alveolar adenocarcinoma cells. Replication efficiencies might link with the notable mutations in viral polymerase complex genes PB2 and PA. Heterogeneous virulence that the viruses displayed in cells and mice may not be linked with the human disease; however, it provides a background to understand the differences in symptomatology, immune responses, and viral dynamics of clinically relevant cases. The study mandates the more comprehensive analysis of 2009 pandemic influenza H1N1 strains and the factors which might be responsible for a different phenotypic behavior in humans.

## Methods

### Viral strains

A total of three pandemic Influenza H1N1 strains, namely A/Nanchang/8002/2009 (NC2), A/Nanchang/8008/2009 (NC8), A/Nanchang/8011/2009 (NC11), were used for *in vitro* and *in vivo* studies. All were isolated from nasopharyngeal (NP) swabs of adult patients who were admitted to a local hospital in Nanchang, Jiangxi province of China, in December 2009. Samples were collected before initiation of virological treatment in each case. These patients had similar courses of infection in terms of viral shedding and disease severity. They had no underlying illnesses (Table 1). All patients eventually recovered. Viral isolation was attained in 9- to 11-day-old embryonated eggs as described previously [38] with the exception of incubation at 33 °C. Samples with hemagglutination titer > 1:2 were considered positive and further confirmed by real time RT-PCR for pdm H1N1 virus using pandemic H1N1 influenza diagnostic kit (Liferiver, Shanghai, China) based on World Health Organization and US CDC protocol [39]. The viral stocks were further titrated by egg infectious dose_50_ (EID_50_) and used for *in vitro* and *in vivo* assays without further passage.

### Sequencing

Whole viral genome sequencing was performed for each strain. RNA were extracted from NP swabs using Trizol (invitrogen) followed by reverse transcription by high-capacity cDNA RT kit (Applied Biosystems, Foster City, USA) and PCR using primers specific for each viral gene segment. Purified PCR preps (Promega, Madison, USA) were sequenced from Invitrogen (Guangzhou, China). Sequences were aligned and assessed by ClustalW multiple alignment tools. Comparisons were made with the prototype strains A/California/04/2009 and A/California/07/2009.

### Infection in human adenocarcinoma alveaolar epithelial (A549) cells

An *in vitro* infection model was developed in adenocarcinoma human alveolar epithelial cells (A549) (ATCC, USA). Briefly, A549 cells, freshly seeded in 24-well plates, were infected with three different strains of pdm H1N1 (NC2, NC8, NC11) at MOI 2 in *v*HAM’s F12 medium (M & C Gene Technology) containing 1 μg/ml of TPCK trypsin. MOI was calculated by EID_50_ titers. After 2 hrs of adsorption, cell supernatants were replaced with fresh medium followed by incubation at 37 °C. Similar treatment with the exception of virus was provided to uninfected cells (blank). Each point was performed in six replicate wells and the experiment was repeated thrice.

For kinetic studies, samples were collected at different time points such as 8 h post infection, 1 day post infection (d.p.i.) and 2 d.p.i.. In the case of the viral loads, supernatants were collected and titrated in MDCK cells (ATCC). For the determination of immune mediators, RNA was extracted using the SV total RNA isolation system (Promega) and reverse transcribed with the high-capacity cDNA RT kit (Applied Biosystems, Foster City, USA) followed by amplification using SYBR Green master mix (Invitrogen). Relative gene expression was calculated after normalization with human *β-actin* gene.

### Confocal laser fluorescent microscopy

A549 Cells seeded on 24-well plates containing cover glass were infected with viral strains at MOI 2 for 1 h at 37 °C followed by washing with HEPES (sigma) thrice and the addition of *v*HAMF12 medium (with no TPCK-trypsin). Cells were incubated at 37 °C for different time intervals, fixed with 2% paraformaldehyde and blocked with 5% bovine serum albumin (BSA) (Sigma). Viral staining was performed with influenza A nucleoprotein antibody (southern biotech) for 16 h at 4 °C. Alexa fluor 555 goat anti mouse IgG (H + L) (Beyotime) diluted 1: 500 in PBS containing 0.05% Tween20 and 3% BSA was used as a secondary antibody while cells were stained for DNA using 4^′^,6, diamino-2-phenylindole (DAPI) (Sigma) diluted 1:1000 in PBS. Slides were observed by confocal laser fluorescence microscope (Olympus Fluoview FV1000). Data is the representative of three independent experiments.

### Animal experiments

Female C57/BL6 and BALB/C mice (8–10 weeks of age) were obtained from Vital River Laboratory (Beijing, China) and maintained on a standard animal diet in a SPF facility with controlled temperature and humidity. Initially, to compare the virulence and pathogenesis of viral strains (NC2, NC8, NC11), C57/BL6 mice (n = 10) were intranasally infected with 10^5^ EID_50_ in a final volume of 50 μl. NC8 infection at a higher dose of 10^6^ EID_50_ was further compared with NC2 and NC11 due to non-lethal infection. To investigate the detailed virulence profile of pdm H1N1 strains, MLD_50_ experiments were set up in C57/BL6 and BALB/C mice. Animals were grouped (n = 10) and infected with 10-fold diluted pdm H1N1 influenza strains ranging from 10^6^ to 10^3^ per mouse. Mock infection with HBSS was given to healthy controls. Animals were observed daily for weight loss and mortality up to 14 d.p.i.. A loss of more than 20% in original body weight was considered the humane end point for mortality.

### Viral loads

Three animals from each group were euthanized at days 0, 1 and 3 p.i. and their organs collected, *i.e.*, lungs, liver and brain. Organ homogenates were prepared in vDMEM (10% w/v) and assayed for viral loads in MDCK cells with the detection limit of 10 TCID_50_/ml as described previously [14].

### Histopathology

On days 0, 1 and 3, p.i. animals were euthanized, and lung tissues were removed and fixed in 10% buffered formalin. Fixed tissues were processed for paraffin wax-embedded sectioning and 5 μm thin sections were stained with hematoxylin and eosin (H & E) and observed; pictures were taken using a Nikon Eclipse 80i microscope (Nikon).

### Measurement of cytokines by quantitative PCR (qPCR)

For the measurement of host immune response, lungs of virus-infected animals (n = 5/group) were collected in an RNAlater (Ambion Inc) at different time intervals. Expression of immune response genes was studied by real time qPCR performed with 0.5pmol/μl of forward and reverse primers targeting the gene of interest. Reactions were run in duplicate, and mean values were normalized with β-actin gene expression. Primer pairs and PCR conditions will be provided upon request.

### Statistics

Statistical analyses were performed using PAWS Statistics 18 (SPSS Inc., Chicago, IL, USA). Fisher’s exact and Chi square tests were used for comparison of categorical data, and the two-tailed t-test was applied in cases of continuous variables. Survival analyses were performed by the Kaplan-Meier method and significant differences were measured by log-rank test. Contingency analysis was applied to assess the number of survivors in each group. Significant differences in viral loads, cytokine measurement, and weight loss and hazard ratios were analyzed by Student’s t-test.

### Ethics statement

This study was approved by the ethical committees of Shantou University Medical College, Shantou, China (permit number SUMC 2011–058) and Infectious Disease Hospital, Nanchang University, Nanchang 9th Hospital (permit number 2009–02). Written consents were obtained from all participants involved in the study.

## Competing interests

The authors declare that they have no competing interests.

## Authors’ contributions

DJK, AF, AL, YL and SR conceived the study and designed the experiments. GL, ZZ, WD, RT, AL and AF performed the experiments. YL, PW, JH and JL contributed in sample collection. AF, AL and DJK analyzed the data and wrote the manuscript. All authors read and approved the final manuscript.

## Supplementary Material

Additional file 1 **Comparison of weight loss kinetics in different strains of pandemic influenza H1N1 Viral strains; NC2, NC8 and NC11 was analyzed in C57/BL6 mice (n = 10/group)**. Viral infection was established with 105 EID50 of NC2, NC11 and 106 EID50 of NC8 and animals were observed up to 14 days. Weight losses in each animal infected with NC2 (a), NC8 (b) and NC11 (c). Significant differences were observed at 3 dpi, NC8 vs NC2 (*P* < 0.0001), NC8 vs NC11 (0.0007). Click here for file

Additional file 2 **Comparison of host immune response between A/Nanchang/8002/2009 infected C57/BL6 and BALB/C mice.** Kinetics of host immune response was observed in C57/BL6 mice infected with 105 EID50 of A/Nanchang/8002/2009 H1N1 (NC2). Animals were euthanized at 0, 1 and 3 dpi. Lung mRNA levels of major proinflammatory markers were determined by real time RT-PCR. Results are presented as mean ± SEM of mRNA levels normalized with mouse β-actin gene. Statistical differences were calculated by Mann Whitney U test. *** - *P* <0.0001, ** - *P* < 0.001 , * - *P* < 0.01. Click here for file

Additional file 3 Mutation analyses of selected strains of pdm H1N1 influenza virus. Click here for file
