# Supplementary material for: Heterogeneous virulence of pandemic 2009 influenza H1N1 virus in mice
Source: Virol J. 2012 Jun 6;9:104. doi: 10.1186/1743-422X-9-104 (PMC3444956; doi:10.1186/1743-422X-9-104)
Supplement: Additional file 1 — Comparison of weight loss kinetics in different strains of pandemic influenza H1N1 Viral strains; NC2, NC8 and NC11 was analyzed in C57/BL6 mice (n = 10/group). Viral infection was established with 105 EID50 of NC2, NC11 and 106 EID50 of NC8 and animals were observed up to 14 days. Weight losses in each animal infected with NC2 (a), NC8 (b) and NC11 (c). Significant differences were observed at 3 dpi, NC8 vs NC2 (P < 0.0001), NC8 vs NC11 (0.0007). [file 1743-422X-9-104-S1.pdf]

Additional file 1

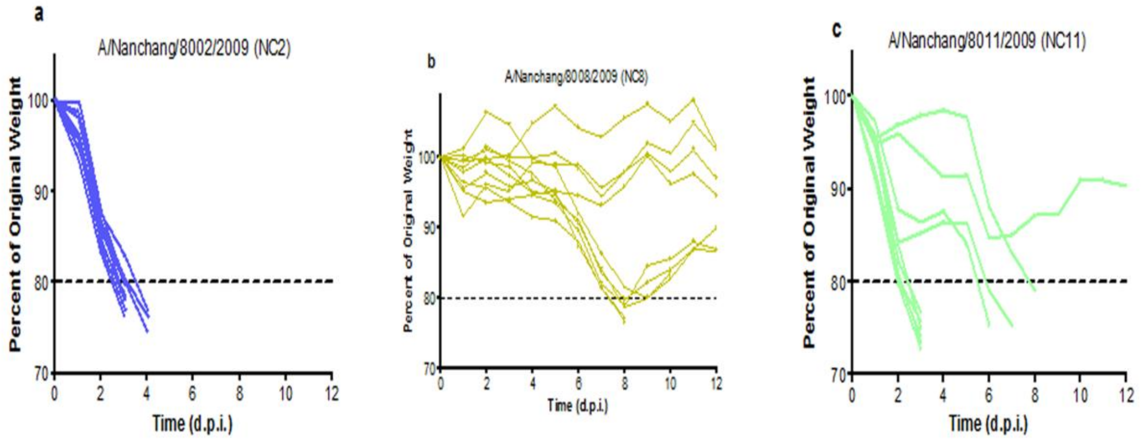

Comparison of weight loss kinetics in different strains of pandemic influenza H1N1 Viral strains; NC2, NC8 and NC11 was analyzed in C57/BL6 mice (n = 10/group). Viral infection was established with 10^5 EID_{50} of NC2, NC11 and 10^6 EID_{50} of NC8 and animals were observed up to 14 days. Weight losses in each animal infected with NC2 (a), NC8 (b) and NC11 (c). Significant differences were observed at 3 dpi, NC8 vs NC2 ($P < 0.0001$), NC8 vs NC11 (0.0007).
