# Supplementary material for: Heterogeneous virulence of pandemic 2009 influenza H1N1 virus in mice
Source: Virol J. 2012 Jun 6;9:104. doi: 10.1186/1743-422X-9-104 (PMC3444956; doi:10.1186/1743-422X-9-104)
Supplement: Additional file 2 — Comparison of host immune response between A/Nanchang/8002/2009 infected C57/BL6 and BALB/C mice. Kinetics of host immune response was observed in C57/BL6 mice infected with 105 EID50 of A/Nanchang/8002/2009 H1N1 (NC2). Animals were euthanized at 0, 1 and 3 dpi. Lung mRNA levels of major proinflammatory markers were determined by real time RT-PCR. Results are presented as mean ± SEM of mRNA levels normalized with mouse β-actin gene. Statistical differences were calculated by Mann Whitney U test. *** - P <0.0001, ** - P < 0.001 , * - P < 0.01. [file 1743-422X-9-104-S2.pdf]

Additional file 2

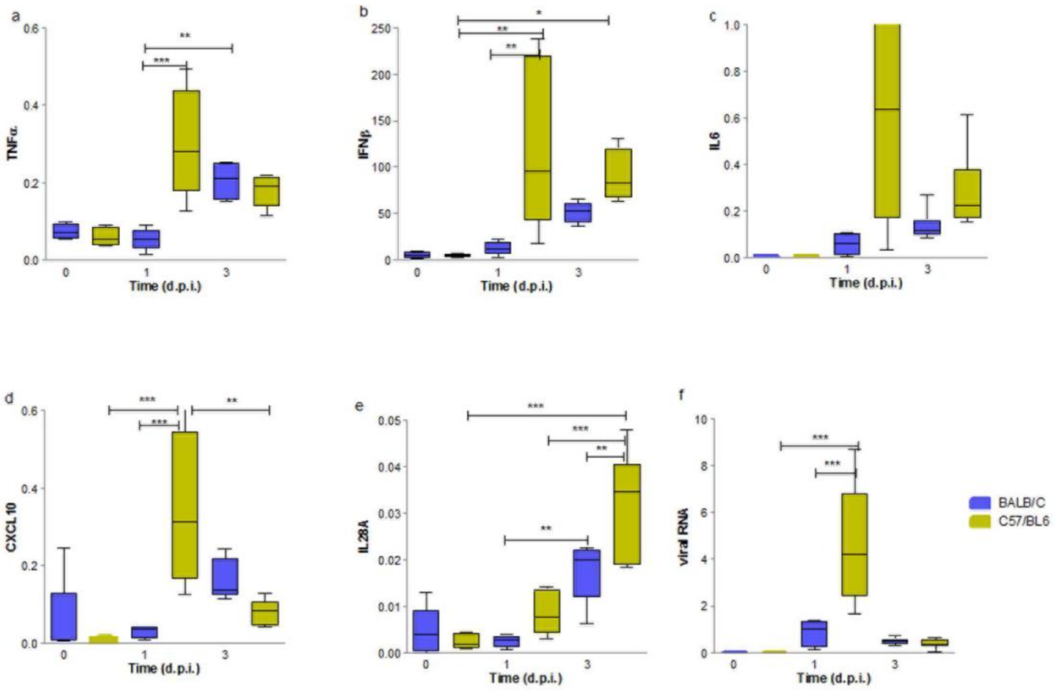

Comparison of host immune response between A/Nanchang/8002/2009 infected C57/BL6 and BALB/C mice

Kinetics of host immune response was observed in C57/BL6 mice infected with 10^5 EID_{50} of A/Nanchang/8002/2009 H1N1 (NC2). Animals were euthanized at 0, 1 and 3 dpi. Lung mRNA levels of major proinflammatory markers were determined by real time RT-PCR. Results are presented as mean ± SEM of mRNA levels normalized with mouse β-actin gene. Statistical differences were calculated by Mann Whitney U test. *** - P <0.0001, ** - P < 0.001 , * - P < 0.01
