# Supplementary material for: Heterogeneous virulence of pandemic 2009 influenza H1N1 virus in mice
Source: Virol J. 2012 Jun 6;9:104. doi: 10.1186/1743-422X-9-104 (PMC3444956; doi:10.1186/1743-422X-9-104)
Supplement: Additional file 3 — Mutation analyses of selected strains of pdm H1N1 influenza virus. [file 1743-422X-9-104-S3.pdf]

Additional file 3

Mutation analysis of selected strains of pdm H1N1 influenza virus

		A/Nanchang/8002/2009 (NC2)	A/Nanchang/8008/2009 (NC8)	A/Nanchang/8011/2009 (NC11)
Sample collection		Dec 9 2009	Dec 19 2009	Dec 22 2009
Age (yrs)		27	15	33
Gender		M	F	F
Temp. (^{o}C)		39.4	38.5	NA
Hospitalization		✓	✓	✓
Complication				
Pregnancy/ other Co-morbidities				
**HA**	Genbank ID	CY089608	CY089616	CY089624
	K71N			
	P100S	✓	✓	✓
	D144E			
	S145P		✓	✓
	I208L			✓
	T214A			
	S220T	✓	✓	✓
	I338V	✓	✓	✓
	S340F			
	E391K	✓		
	A409V	✓		✓
	H416P			
**NA**	Genbank ID	CY089610	CY089618	CY089626
	N42S			
	V106I	✓	✓	✓
	N248D	✓	✓	✓
NP	Genbank	CY089609	CY089617	CY089625
	V100I	✓	✓	✓
	D101N			✓
M2	Genbank ID	CY089611	CY089619	CY089627
	C321G	✓	✓	✓
NS1	Genbank ID	CY089612	CY089620	CY089628
	I123V	✓	✓	✓
	I156V	✓		
	N205S			✓

mutations are compared with prototypic strain: A/California/07/2009 H1N1, NA – not available, ND – Not done
